# Protocol for isolating human extracellular vesicles from nephrotic urine and enriching autoimmunoglobulin-triggered extracellular vesicles

**DOI:** 10.1016/j.xpro.2026.104661

**Published:** 2026-07-02

**Authors:** Karen Lahme, Catherine Meyer-Schwesinger

**Affiliations:** 1 Institute of Cellular and Integrative Physiology, Center for Experimental Medicine, University Medical Center Hamburg-Eppendorf (UKE), 20246 Hamburg, Germany; 2 Hamburg Center of Kidney Health, University Medical Center Hamburg-Eppendorf (UKE), 20246 Hamburg, Germany

**Keywords:** Cell Biology, Health Sciences, Clinical Protocol

## Abstract

This protocol describes the isolation of total urinary extracellular vesicles (EVs) from nephrotic patient urines and enrichment of AutoImmunoglobulin-Triggered Extracellular Vesicles (AIT-EVs). Procedures for urine collection, sample processing, buffer preparation, antibody coupling to DynaBeads, urine ultrafiltration, and total EV isolation are provided. The protocol further details EV concentration measurement and selective AIT-EV capture and elution using anti-human IgG4 antibodies. This approach enables reproducible enrichment of AIT-EVs and can be adapted to isolate other EV subtypes by substituting the capture antibody.

## Highlights

• Steps for isolating urinary extracellular vesicles from nephrotic patient urine
• Instructions for enriching AIT-EVs from MN patient urine
• Guidance on immunoprecipitation of specific EV subtypes using capture antibodies
• Steps for preparing AIT-EVs for downstream molecular and functional analyses

## Abstract (editor highlights)

Publisher’s note: Undertaking any experimental protocol requires adherence to local institutional guidelines for laboratory safety and ethics.

## Graphical abstract

**Figure:**
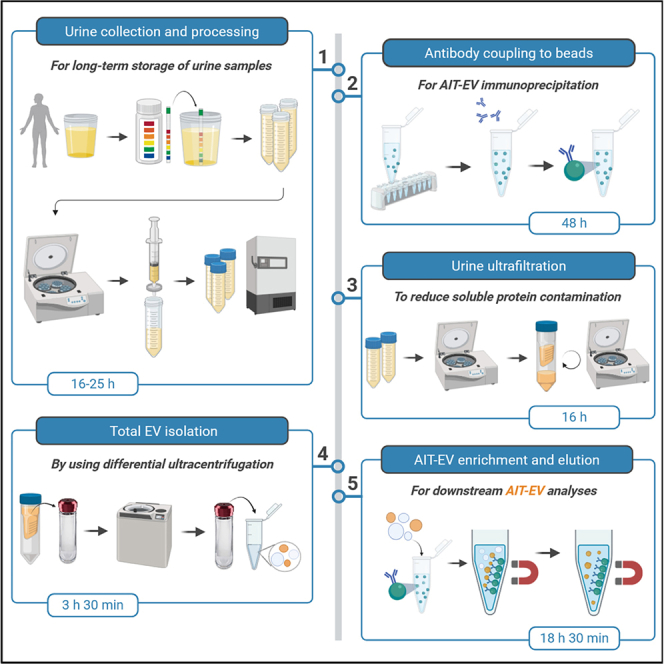

## Before you begin

This protocol describes the enrichment of **A**uto**I**mmunoglobulin-**T**riggered **E**xtracellular **V**esicles (AIT-EVs) from total urinary extracellular vesicles (EVs) isolated from human urine samples. The workflow includes urine preprocessing, isolation of total urinary EVs, and subsequent enrichment of AIT-EVs. It is optimized for samples from nephrotic patients, which are characterized by high protein content and increased EV abundance. To minimize interference from proteinuria during EV isolation and downstream analyses, the protocol incorporates preprocessing and purification steps that reduce protein contamination and improve EV recovery.

### Innovation

This protocol introduces a robust workflow for isolating and enriching AIT-EVs from human urine, including samples from nephrotic membranous nephropathy (MN) patients. While existing methods allow for total urinary EV isolation, they often lack specificity for vesicle subtypes of pathological relevance. Our approach combines standardized urinary EV isolation with an adaptable immunoprecipitation strategy that selectively captures AIT-EVs using specific antibodies.

The method provides several key advancements over conventional protocols: (1) Subtype-specific EV enrichment: Enables targeted isolation of AIT-EVs, implicated in MN pathogenesis and difficult to study using standard EV isolation methods; (2) Optimization for nephrotic urine: Accommodates high contaminating protein loads that are characteristic in nephrotic urine and interfere with conventional EV capture and analyses; (3) Adaptable immunoprecipitation workflow: Applies the same strategy to other urinary EV subtypes by substituting the capture antibody, allowing flexibility for different research questions; and (4) Downstream analysis compatibility: Generates enriched AIT-EVs suitable for molecular, functional, and biomarker analyses, enabling translational studies in kidney disease.

By providing a standardized, versatile, and EV subtype-focused approach, this protocol facilitates precise interrogation of pathologically relevant urinary EVs, overcoming limitations of existing methods that primarily isolate total EV populations without subtype specificity.

### Institutional permissions

The presented human sample research complies with all relevant ethical regulations. Written consent by the patients and ethics approval were obtained for the prospective urine analyses and correlations to diagnostic biopsy samples of nephrotic patients (2023-101080-BO-ff).

This protocol describes the processing and long-term storage of 1 L urine collected from MN patients, followed by enrichment of AIT-EVs from 100 mL frozen urine aliquots. All volumes and timing in this protocol are optimized for these sample sizes.

### Workflow overview

**Figure 1:**
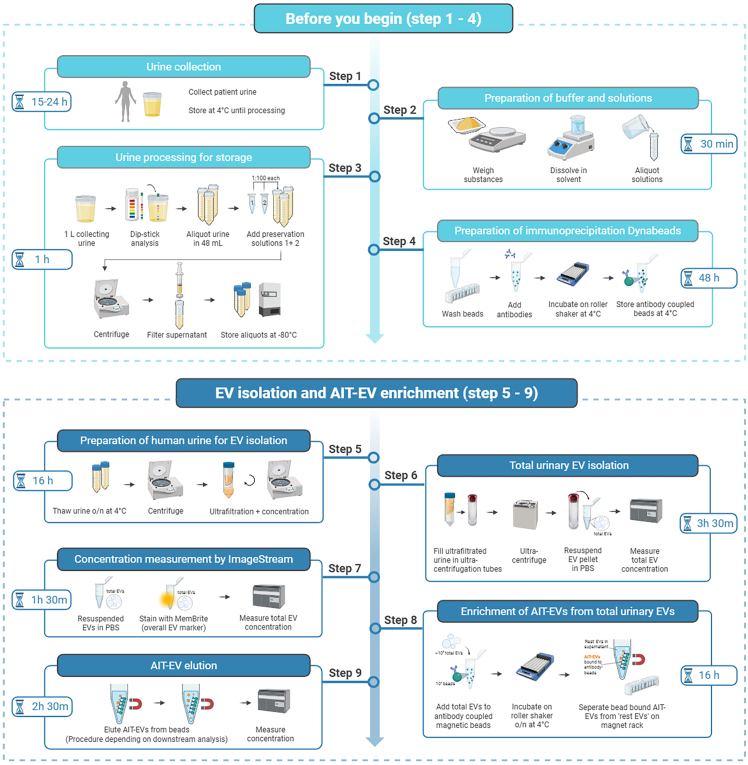
Detailed workflow timeline Steps 1 – 4 preparations before you begin. Steps 5 – 9 urine processing for total urinary EV isolation and AIT-EV enrichment.

The overall workflow of the protocol consists of the following main steps (Figure 1).

1. Collect patient urine.
2. Prepare EV Preservation Solutions 1 and 2 for urine processing and buffers and solutions needed for AIT-enrichment workflow.
3. Aliquot urine, supplement with EV Preservation Solutions, process, and store at −80°C.
4. Prepare anti-human IgG4 antibody-coupled DynaBeads for immunoprecipitation.
5. Thaw and pre-process urine aliquots and reduce protein contamination by ultrafiltration.
6. Isolate total urinary EVs by differential ultracentrifugation.
7. Measure total EV concentration by ImageStream.
8. Enrich AIT-EVs by immunoprecipitation using antibody-coupled beads.
9. Elute enriched AIT-EVs for downstream analyses.

#### Overall timing

Urine collection and processing for long-term storage: 16–25 hours.

Antibody coupling to DynaBeads: 48 hours.

From preparation of thawed urine samples for EV isolation to AIT-EVs ready for downstream analyses, expect a total duration of 3 days.

### Preparation of buffers and solutions

**Timing: 30 min**

10. EV Preservation Solution 1: 600 mM NaN_3_/200 mM ethylene glycol-bis(β-aminoethyl ether)-N,N,N′,N′-tetraacetic acid (EGTA).
  a. For 100 mL, weigh 4.29 g of NaN_3_ and 7.61 g of EGTA and dissolve both together in 80 mL of ultrapure water.
  b. Wait until everything is fully dissolved, then fill up the volume to 100 mL with ultrapure water.
  c. Make aliquots of 10 mL and store at −20°C, avoid freeze-thaw cycles.

**CRITICAL:** Sodium azide (NaN_3_) is hazardous. Perform all steps under a fume hood while wearing appropriate personal protective equipment. Clearly label aliquots with hazard symbols.

11. EV Preservation Solution 2: 100 mM phenylmethylsulfonyl fluoride (PMSF).
  a. For 100 mL, weigh 1.75 g of PMSF and dissolve in 80 mL of pure ethanol.
  b. Wait until everything is fully dissolved, then fill up the volume to 100 mL with pure ethanol.
  c. Make aliquots of 10 mL and store at −20°C, avoid freeze-thaw cycles.

***Note:*** Recipes for both solutions can be scaled as needed, solutions can be stored at −20°C for up to 3 months. Avoid freeze-thaw cycles.

**CRITICAL:** Phenylmethylsulfonyl fluoride (PMSF) is hazardous. Perform all steps under a fume hood while wearing appropriate personal protective equipment. Clearly label aliquots with hazard symbols.

12. Coupling Buffer: 100 mM sodium phosphate pH 8.0.
  a. For 100 mL, weigh 1.64 g of NaH_2_PO_4_ H_2_O and dissolve in 80 mL of ultrapure water.
  b. Adjust pH to 8.0 at 22–25°C using NaOH.
  c. Fill up to 100 mL with ultrapure water.
13. Storage Buffer: PBS pH 7.4 / 2 mM EDTA.
  a. For 100 mL, combine 10 mL of 10 x PBS (pH 6.8) and 10 mL of 20 mM EDTA (pH 7.4) in a measuring cylinder.
  b. Add ultrapure water to bring the total volume to 100 mL.

### Preparation of human urine samples

**Timing: Variable 16–25 h (per 1 L of urine)**

***Note:*** To minimize pre-analytical variability, collect urine samples using a standardized clinical procedure.

14. Human urine sample collection.
  a. Provide patients with a sterile screw-cap urine collection container.
  b. Instruct patients to collect urine continuously during the day and overnight until the following morning.
    **CRITICAL:** Ideally 24-h urine collections are performed. For this, the patient chooses a start time, for example 7:00 in the morning. At the start time, the bladder is emptied into the toilet and this urin is discarded. This first void is ***not*** collected and marks the start of the 24-h collection period. From then on, every drop of urine is collected for the next 24 h in the collection container. This includes urine during the day, evening, and night. Urine samples should ideally be maintained at 4°C throughout the entire 24-h collection period to preserve EVs.^1^^,^^2^ At exactly the same time the next day, for example 7:00 the following morning, the bladder is emptied one final time and this urine is added to the container. This final void **is part** of the collection.
    ***Note:*** Urine samples should ideally be maintained at 4°C throughout the entire 24-h collection period to preserve EVs.^1^^,^^2^ However, continuous cooling during collection may not always be feasible in routine clinical settings. Therefore, immediately after completion of the collection period, urine samples should be transferred to 4°C.
  c. Retrieve urine samples from 4°C storage between 9:00 and 11:00 a.m and start with urine processing for long-term storage immediately.
    **CRITICAL:** Following completion of the collection period, urine samples should be processed as quickly as possible and within 4 h after picking up to preserve EV integrity.
    ***Note:*** Collection of diagnostic urine is performed by patients as part of routine clinical procedures. Total collection periods may vary between 15 and 24 h, depending on hospitalization time and individual commencement of treatment. Collection periods should always include an overnight interval.
    **CRITICAL:** All experiments involving human urine samples require prior ethical approval and written informed consent from the patients.
15. Human urine sample preparation for storage.
  ***Note:*** Process urine samples promptly and as quickly as possible and no later than 4 h after the collection process is finished to preserve EV integrity.^1^^,^^2^^,^^3^
  a. Mix the urine collection container by inverting and shaking thoroughly.
  b. Aliquot the urine sample before proceeding. Prepare enough sterile 50 mL tubes for your sample size. So, for 1 L of urine, you would need 20 × 50 mL tubes.
  c. Transfer 49 ml of urine into each tube.
    ***Note:*** Adjust aliquot volumes to experimental needs to avoid repeated freeze–thaw cycles, which reduce EV stability and recovery. If you are able to process larger or smaller aliquots it is better to adjust the aliquot size now to your needs. See also the recommendations guided by the International Society of Extracellular vesicles (ISEV) on urine handling and storage.^2^
  d. Perform urine dipstick analysis to document basic urine parameters. Dip the strip into urine, remove excess liquid on tissue paper.
  e. Incubate dipstick for 2 min.
  f. Photograph the dipstick and document all parameters to assess urine status including proteinuria and bacterial infection.
    ***Note:*** Record urine dipstick results such as protein, nitrite, pH, specific gravity, leukocytes and erythrocytes values and relevant clinical data from the doctor’s letter,^1^ like serum albumin, creatinine, triglycerides, cholesterol and autoantibody titer, as well as urine creatinine, proteinuria, albuminuria, urea nitrogen from the day of collection to support downstream interpretation as these parameters may influence EV abundance and contamination.
  g. Add 0.5 mL of EV Preservation Solution 1 and 2 each (that corresponds to a 1:100 dilution).
  h. Centrifuge the urine at 2,500 × *g* for 10 min at 4°C using a pre-cooled swinging-bucket rotor in Eppendorf 5804 R Centrifuge.
  i. Carefully transfer the supernatant into a 50 mL syringe fitted with a 5 μm filter and filter into a new sterile 50 mL tube. Discard the pellet.
    **CRITICAL:** Avoid disturbing the urine pellet, which contains cells, debris, and bacteria. Transfer the supernatant in a single continuous step.
  j. Store urine aliquots at −80°C.

### Preparation of immunoprecipitation beads

**Timing: 49 h**

***Note:*** Since the coupling procedure includes a 48 h incubation period you should start 2 days before you start the EV isolation.

***Note:*** Always use freshly prepared Coupling and Storage Buffer.

***Note:*** Based on our experience, you can expect to obtain approximately 10^6^ to 10^9^ total EVs out of 100 mL of MN patient urine. From healthy control urine approximately 10^4^ to 10^7^ total EVs are to be expected.

Depending on the sample, 1 × 10^7^ DynaBeads and 4 μg anti-human IgG4 antibody are typically sufficient to isolate AIT-EVs from around 1 x 10^9^ total EVs. You may need to adjust the number of DynaBeads and the amount of antibody to your specific experimental setup.

16. Coupling of antibodies to magnetic DynaBeads.
  ***Note:*** Prepare one tube of coupled DynaBeads per 100 mL of urine sample.
  a. Vortex the DynaBead suspension for 1 min to obtain a homogenous solution.
  b. Transfer 1 × 10^7^ DynaBeads (25 μL) into a 1.5 mL tube and add an equal volume (25 μL) of Coupling Buffer. Mix by vortexing.
  c. Place the tube on a magnetic rack until you clearly see the DynaBeads gathering on the side of the tube facing the magnet. This usually occurs within seconds.
  d. Remove the supernatant as long as the tube is still placed in the rack, don’t disturb the DynaBeads.
  e. Remove the tube from the magnetic rack.
  f. Add 50 μL Coupling Buffer, mix by vortexing briefly.
  g. Repeat steps c to e as additional washing step.
  h. Add 350 μL of Coupling Buffer and resuspend by vortexing briefly.
    **CRITICAL:** Do not use less than 350 μL of Coupling Buffer to ensure proper mixing during the 48 h incubation in Step j.
  i. Add 4 μg of antibody. For human IgG4 (concentration = 0.5 mg/mL) that is 8 μL of antibody solution.
  j. Incubate for 48 h at 4°C on a roller shaker.
    ***Note:*** Do not allow the DynaBead–antibody suspension to settle. Make sure it is mixed by continuous agitation during incubation on the roller shaker.
    ***Note:*** The coupling procedure can also be conducted at 22–25°C or 37°C, which accelerates the coupling process but also depends on the stability of the used antibody. Refer to the manufacturer‘s instructions for further information (see the DynaBeads manual).
17. Preparing the coupled DynaBeads for storage.
  a. After incubation, take tubes from the shaker and place them on a magnetic rack. Discard the supernatant carefully without disturbing the DynaBeads.
  b. Remove tubes from the rack, add 500 μL of Storage Buffer, mix gently.
  c. Place tubes back on the rack, discard supernatant carefully.
  d. Remove tube and add 500 μL of Storage Buffer.
  e. Store antibody-coupled DynaBeads at 4°C.

## Key resources table

**Table:** 

REAGENT or RESOURCE	SOURCE	IDENTIFIER
**Antibodies**		

Actinin-4, alpha (host rabbit) (Immunoblot 1:1000) (Immunofluorescence: 1:100)	ImmunoGlobe	IG-701; Cat#0042-05; RRID:AB_2490474
Annexin A1 (host rabbit) (Immunoblot 1:2000) (ImageStream 1:100) (Immunofluorescence: 1:400)	Abcam	Cat#ab214486; RRID:AB_2890907
Annexin V, CoraLight plus 647 (host mouse) (ImageStream 1:100)	ProteinTech	Cat#CL647-66245; RRID:AB_2920278
ATPB (host mouse) (Immunoblot 1:1000)	Abcam	Cat#ab14730; RRID:AB_301438
CD9 (host rabbit) (Immunoblot 1:1000)	Abcam	Cat#ab92726; RRID:AB_10561589
CD63 (host rabbit) (Immunoblot 1:1000)	Abcam	Cat#ab216130; RRID:AB_3076642
CD63, Pacific Blue (host mouse) (ImageStream 1:50)	BioLegend	Cat#BLD-353012; RRID:AB_10918981
CD81, Pacific Blue (host mouse) (ImageStream 1:50)	BioLegend	Cat#BLD-349516; RRID:AB_2687127
Flotillin (host mouse) (Immunoblot 1:1000)	BD Bioscience	Cat#610821; RRID:AB_398140
Human IgG4 (H&L), CoraLite 488 (host mouse) (ImageStream 1:100) (Immunofluorescence: 1:100)	ProteinTech	Cat#CL488-66408; RRID:AB_2883328
Human IgG4 (H&L), CoraLite 647 (host mouse) (ImageStream 1:100)	ProteinTech	Cat#CL647-66408; RRID: AB_2920282
Human IgG4 (H&L), (host mouse) (Immunoprecipitation 4 μg per 1 x 10^7^ Dynabeads	Southern Biotech	Cat#9190-01; RRID:AB_2796681
Human IgG4, Fc-HRP (host mouse) (Immunoblot 1:10000)	SouthernBiotech	Cat# 9200-05; RRID:AB_2796691
Lamp2 (host rabbit) (Immunoblot 1:1000)	Sigma	Cat#L0668; RRID:AB_477154
Limp2 (host rabbit) (Immunoblot 1:1000) (Immunofluorescence: 1:500)	Paul Saftig, CAU Kiel, Germany	N/A
Lmp7 (host rabbit) (Immunoblot 1:1000)	Abcam	Cat#ab3329; RRID:AB_303708
Mitofusin-2, D2D10 (host rabbit) (Immunoblot 1:1000) (Immunofluorescence: 1:200)	Cell Signaling	Cat#9482; RRID:AB_2716838
MnSOD (host rabbit) (Immunoblot 1:1000)	Millipore	Cat#06-984; RRID:AB_310325
Nephrin (host guinea pig) (Immunoblot 1:1000) (Immunofluorescence: 1:200)	Progen	Cat#GP-N2; RRID:AB_2904121
PLA_2_R1 (host rabbit) (Immunoblot 1:1000)	Atlas	Cat#HPA012657; RRID:AB_2299684
Podocin (host rabbit) (Immunoblot 1:1000)	Sigma	Cat#P0372; RRID:AB_261982
Synaptopodin (host guinea pig) (Immunoblot 1:1000)	Synaptic Systems	Cat#163004; RRID:AB_10549419
THSD7A (host rabbit) Immunoblot 1:1000)	Atlas	Cat#HPA000923; RRID:AB_1080271
TSG101, EPR7130(B) (host rabbit) (Immunoblot 1:1000)	Abcam	Cat# ab125011; RRID:AB_10974262
Ubiquitin, linkage-specific K48 (host rabbit) (Immunoblot 1:1000) (Immunofluorescence: 1:300)	Abcam	Cat# ab140601; RRID:AB_2783797
UCH-L1 (host rat) (Immunoblot 1:250)	Sosna et al. 2013^4^	N/A
14-3-3, AF546 (host mouse) (ImageStream 1:100)	Santa Cruz	Cat#sc-1657 AF546; pan 14-3-3 H8; RRID:AB_626618
14-3-3 (host mouse) (Immunoblot 1:1000)	Santa Cruz	Cat#sc-1657; pan 14-3-3 H8; RRID:AB_626618

**Biological samples**		

Patient diagnostic urine	Nephrology Asklepios Klinikum Barmbek	N/A

**Chemicals, peptides, and recombinant proteins**		

Dynabeads™; M-450 Tosylactivated	Thermo Fisher	Cat#14013
Sodium dihydrogen phosphate monohydrate	Fluka	Cat#71506
Sodium azide	Sigma	Cat#S2002
PMSF	Sigma	Cat#P7626
EGTA	Carl Roth	Cat#3054.2
TRIS	Carl Roth	Cat#AE15.4
EDTA	Sigma	Cat#E9884
Glycine	Carl Roth	Cat#3790.2

**Critical commercial assays**		

LysoView 488	Biotium	Cat#77067
MemBrite® Fix 594/615 (Mb594)	Biotium	Cat#30096
MitoSOX®	Invitrogen	Cat#M36008

**Deposited data**		

AIT-EV proteomes PXD049008	PRIDE	reviewer_pxd049008@ebi.ac.uk

**Software and algorithms**		

AIT-EV-DetectionAndAnalysis	Lahme, K. et al.^5^; Mendeley Data	Code available at https://doi.org/10.17632/bmykmvv6cz.1
Amnis IDEAS software (version 6.2)	Cytek Biosciences	N/A
Amersham ImageQuant 800	Cytiva	N/A
BioRender	BioRender	https://app.biorender.com
GraphPad Prism 8.2.1 for Mac OS	GraphPad	www.graphpad.com
HiD® HistoDigital reconstruction	Gaffling et al. 2015^6^	https://www.ncbi.nlm.nih.gov/pubmed/25312918
ImageSPViewer	Tröndle	N/A
Image Viewer	JEOL	N/A
NTA 3.0 software	Nanosight	N/A
ZEN 3.0	Zeiss	N/A

**Other**		

Multistix 10 SG	Siemens Healthineers AG	N/A
ROTI®Spin MAXI, 100 kDa (15 ml ultrafiltration tubes)	Carl Roth	Cat# 25TE.2
DynaMag-2 magnet	Invitrogen	Cat#12321D
Roller shaker 6 digital	IKA	Cat#0004011000
Centrifuge 5804 R	Eppendorf	N/A
Ultracentrifuge OPTIMA l-80K	Beckman coulter	N/A
Rotor 70Ti	Beckman coulter	N/A
26.3 mL Polycarbonate Bottle with Cap Assembly, 25 x 89mm	Beckman coulter	Cat#355618
Electron microscope TEM Leo 910	Zeiss	ImagesSPViewer
Electron microscope TEM JEM 1400Plus with tomography high tilt holder	JEOL	TEM-Center Ver. 1.6.19.5342 Serial EM Ver. 4.1.9
Electron microscope SEM JSM 7500F	JEOL	PC-SEM Vers. 3.0.1.28
ImageStream X MkII	ISX Amnis/Millipore/Sigma	RRID: SCR_018589
Leica DMi8 M/C/A inverted microscope using a HC PL Fluotar 20x/0,4 CORR PH1 objective	Leica	N/A
LSM800 with Airyscan 1	Zeiss	N/A
LSM980 with Airyscan 2	Zeiss	N/A
Nanoparticle tracking analyzer LM10 unit	Nanosight	N/A
Nikon Eclipse TiE, Visitron-SD-TIRF with SoRa unit from Yokogawa	Nikon	N/A

## Materials and equipment

### EV Preservation Solutions

**Table:** EV Preservation Solution 1: 660 mM NaN_3_ / 200 mM EGTA

Reagent	MW	Concentration	Dilution in sample	Final concentration in urine sample
Sodium azide	65.01 g/mol	660 mM	1:100	6.6 mM
EGTA	380.35 g/mol	200 mM	1:100	2 mM

Solve in dH_2_O.

**Table:** EV Preservation Solution 2: 100 mM PMSF

Reagent	MW	Concentration	Dilution in sample	Final concentration in urine sample
PMSF	174.19 g/mol	100 mM	1:100	1 mM

Solve in 100 % ethanol.

Prepare 100 mL of each Preservation Solution, dissolve components in solvent as shown and mix on a magnetic stirrer at 22–25°C.

***Note:*** Prepare 10 mL aliquots to avoid several freeze and thaw cycles. Store at −20°C up to 3 months.

### Buffer for antibody-to-DynaBead coupling

**Table:** Coupling Buffer

Reagent	Concentration	Add to 100 mL
Sodium dihydrogen phosphate monohydrate	100 mM	1.64 g

Solve in dH_2_O, adjust pH to 8.0.

To prepare 100 mL of antibody-DynaBead Coupling Buffer, dissolve 1.64 g sodium phosphate in 90 mL dH_2_O on a magnetic stirrer at 22–25°C. Adjust the pH to 8.0 with 0.1 M sodium hydroxide and fill up with dH_2_O to 100 mL. Always use freshly prepared Coupling Buffer.

**Table:** Storage Buffer for antibody-coupled DynaBeads

Reagent	Stock conc.	Dilution	Final conc.	Vol. For 100 mL
PBS pH 6.8	10 ×	1:10	1 ×	10 mL
EDTA pH 7.4	20 mM	1:10	2 mM	10 mL
H_2_O	---	---	---	80 mL

To prepare 100 mL of Storage Buffer, combine 10 mL of 10 x PBS (pH 6.8) and 10 mL of 20 mM ethylenediaminetetraacetic acid (EDTA) (pH 7.4) in a measuring cylinder. Add ultrapure water to bring the total volume to 100 mL. Before you use stock solutions, check them for bacterial growth and visible contamination. Store buffer at 4°C and store up to 2 months.

### Elution Buffer

**Table:** 0.1 M glycine in dH_2_O, pH 2.0

Reagent	MW	Concentration	Add to 100 mL
Glycine	75.07 g/mol	100 mM	0.7507 g

To prepare 100 mL of Elution Buffer, dissolve 0.7507 g glycine in 95 mL dH_2_O on a magnetic stirrer at 22–25°C. Adjust the pH to 2.0 with 1N hydrochloric acid and fill up with dH_2_O to 100 mL. Use always freshly prepared Elution Buffer for experiments.

### Neutralization Buffer

**Table:** 0.5 M tris(hydroxymethyl)aminomethane (TRIS) in dH_2_O, pH 8.0

Reagent	MW	Concentration	Add to 100 mL
TRIS	121.14 g/mol	500 mM	6.057 g

To prepare 100 mL of Neutralization Buffer, dissolve 6.057 g TRIS in 90 mL dH_2_O on a magnetic stirrer at 22–25°C. Adjust the pH to 8.0 with 0.1 M sodium hydroxide and fill up with dH_2_O to 100 mL. Store the buffer at 4°C up to 2 months.

## Step-by-step method details

### Preparation of human urine for EV isolation

**Timing: 16 h**

The following steps and timing are for 100 mL of urine sample, that is two of the previously frozen 50 mL urine aliquots.

***Note:*** Urine samples from nephrotic patients require additional processing compared to samples acquired from healthy patients to reduce contaminating protein content prior to EV isolation.

To minimize protocol-induced variability, process all urine samples, including healthy control samples, using the same workflow.

Based on our experience, you will get 10^6^ to 10^9^ total EVs out of 100 mL of MN patient urine and 10^4^ to 10^7^ total EVs out of 100 mL of healthy control urine.

**Figure 2:**
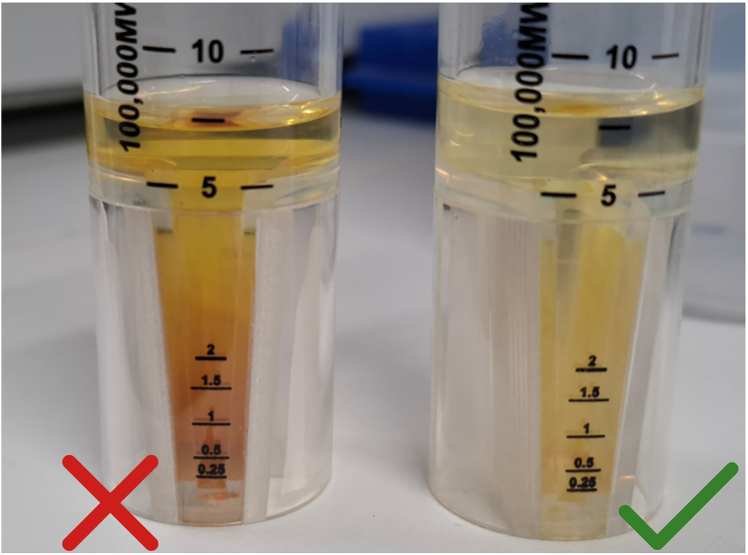
Ultrafiltration of nephrotic urines **Left:** Avoid urine overconcentration during ultrafiltration. Do not allow samples to become brownish and viscous in the ultrafiltration process. **Right:** Successful ultrafiltration resulting in clear, non-viscous urine suitable for EV isolation.
    Therefore, centrifugation time and the number of concentration steps should be adjusted individually for each sample. After every centrifugation step, inspect the sample visually (Figure 2) to prevent overconcentration. Overconcentrated samples frequently show increased protein contamination or artifacts that interfere with downstream analyses, such as smearing in immunoblotting or particle aggregates during nanoparticle tracking analysis (NTA) measurements.
    i. If overconcentration occurs, pipette the concentrated urine out of the filter device into a new tube.
      ***Note:*** Typically, 1–3 ultrafiltration devices are sufficient for processing 100 mL nephrotic urine. However, highly proteinuric, concentrated, or viscous samples may require additional devices due to membrane clogging or overconcentration. Based on our experience, overconcentration risk does not solely correlate with the degree of proteinuria but may also be influenced by additional urine characteristics such as specific gravity and overall urine composition.
    ii. Fill up with PBS to a volume of 15 mL.
    iii. Repeat centrifugation until the 100 mL of starting volume is concentrated to a final sample volume of 30–35 mL. If overconcentration occurs again before you reach the desired final volume, repeat Step i.
  e. Recover the concentrated urine using a pipette and transfer into a sterile tube and proceed immediately with the next step.
    ***Note:*** Ultrafiltration substantially reduces contaminating soluble proteins in nephrotic urine and is essential for reliable downstream EV analyses. Without this step, unfiltered nephrotic urine yields poorly purified EV preparations that are unsuitable for analytical applications. To minimize protocol-induced variability, process all urine samples, including healthy control samples, using the same workflow. We note that ultrafiltration membranes may adsorb a proportion of EVs and thereby reduce total EV recovery. Nevertheless, in nephrotic urine samples this step substantially improves downstream analytical quality by reducing excessive soluble protein contamination.
    Figure 2 left: If the sample becomes viscous and brownish, remove it immediately from the filter unit and do not centrifuge this again. Dilute the concentrate with remaining unconcentrated urine sample or 1x PBS, reload this diluted mixture into a new filter tube, and continue ultrafiltration cautiously.
    Figure 2 right: If the sample remains light-yellow and non-viscous after the centrifugation step, load additional remaining urine and proceed with centrifugation.
    As a general guideline from our experiences, reduce urine volume to approximately one-third of the original volume (e.g., 100 mL can be concentrated to 30-35 mL). Some samples may become overconcentrated before reaching this target volume of 30–35 mL. In such cases, do not continue centrifugation, as this will damage EVs. Instead, dilute the sample with an equal volume of filtered 1× PBS, vortex gently, reload into a new filter unit, and resume centrifugation.
    Note that such samples, even after completion of the ultrafiltration step and dilution with filtered 1× PBS, may still contain elevated levels of residual protein contamination, which should be considered during downstream analyses and may require additional clearing steps or warrant exclusion.

1. Remove aggregates from the samples.
  ***Note:*** Aggregates formed during freezing, including urinary salts and urate crystals, interfere with EV isolation and downstream analyses.^3^^,^^7^
  a. Thaw the urine aliquots overnight at 4°C.
    ***Note:*** Overnight thawing at 4°C is used to allow gradual dissolution of urinary precipitates and is considered to be gentler for EV integrity than thawing faster at room or higher temperature. However, prolonged thawing conditions may influence EV-associated components like RNA integrity or metabolites and should be considered when planning those downstream analyses.
  b. Vortex urine aliquots thoroughly for 1 min.
  c. Centrifuge urine aliquots at 2500 × *g* for 10 min at 4°C using a pre-cooled swinging bucket rotor in an Eppendorf 5804 R centrifuge.
  d. Transfer the supernatant into a new sterile tube and discard the pellet.
2. Ultrafiltration of urine samples.
  a. Transfer 15 mL urine into a 100 kDa ultrafiltration tube (see troubleshooting: problem 1: Ultrafiltration of urine samples).
  b. Place the filter tube in a pre-cooled centrifuge with a swinging bucket rotor and centrifuge in an Eppendorf 5804 R at 2500 × *g* for 10 min at 4°C.
    ***Note:*** Depending on the concentration of the urine, after the first centrifugation step you will have around 1 mL to 7 mL of concentrated urine remaining in the filter device.
  c. Discard the flow through and fill up the remaining urine inside the filter device up to 15 mL with remaining urine sample of the aliquot you are processing.
  d. Repeat centrifugation until the 100 mL of starting volume is concentrated to a final sample volume of 30–35 mL or stop earlier if overconcentration occurs (see Figure 2).
    **CRITICAL:** Urine concentration varies markedly between patients due to differences in renal function, proteinuria, and fluid intake. In nephrotic urine samples, the high protein content necessitates careful reduction of soluble proteins to minimize contamination during EV isolation. However, excessive concentration of the urine sample should be avoided, as this can promote protein aggregation and EV damage.

### Human total urinary EV isolation

**Timing: 3 h and 30 min**

Isolate total urinary EVs from the urine samples by differential ultracentrifugation.

***Note:*** Ensure to follow the general safety and operational guidelines for the ultracentrifuge being used.

**Figure 3:**
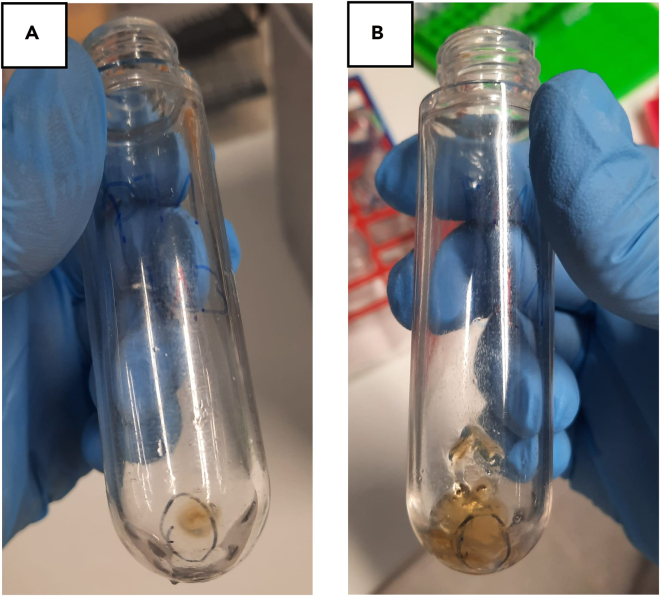
Total urinary EV pellet after ultracentrifugation (A) Smaller EV pellet should be resuspended in 50-100 μl PBS. (B) Larger EV pellet should be resuspended in 300-400 μl PBS.
  k. Transfer 5 μL of EV suspension into a fresh tube and continue with Step 4a for concentration measurement by ImageStream.
  l. Store the remaining EV suspension on ice until proceeding with immunoprecipitation as described in Step 7a.
    **CRITICAL:** It is not recommended to store or freeze the isolated total EVs before immunoprecipitation. Proceed with the workflow without interruption and perform the following AIT-EV immunoprecipitation as soon as possible. Avoiding intermediate storage significantly improves EV quality and integrity.
    ***Note:*** EV numbers reported in the associated manuscript were determined by ImageStream analysis using MemBrite 594/615 as a membrane dye for overall EV labeling. EVs were identified based on side scatter and the MemBrite fluorescence, with MemBrite-positive EVs gated above an intensity threshold of 1 x 10^3^. Absolute EV counts may differ when alternative quantification techniques (e.g., nanoparticle tracking analysis, NTA) are used. Such differences should be considered when normalizing EV input or calculating concentrations for downstream applications when using EV numbers reported in this protocol.

3. Ultracentrifugation.
  a. Divide the ultrafiltered urine from the previous step into two equal parts and transfer each part into a 26.2 mL polycarbonate ultracentrifuge tube.
  b. Fill up each tube to approximately two-thirds of its volume capacity with PBS.
  c. Balance the tubes on a scale to ensure equal weight before ultracentrifugation. Make sure the tubes are properly sealed.
  d. Load tubes into a pre-cooled Ti70 rotor.
  e. Place rotor into a pre-cooled Beckman OPTIMA l-80K ultracentrifuge.
  f. Centrifuge at 100,000 × g for 90 min at 4°C.
  g. Carefully decant the supernatant, then return the tube to an upright position.
  h. Allow the remaining liquid to settle at the bottom for 2 min.
  i. Completely remove the residual supernatant with a pipet.
  j. Resuspend the EV pellet in an appropriate volume of cold 1x PBS (Figure 3) and transfer EV solution into a sterile 1.5 mL tube.
    ***Note:*** Typical resuspension volumes range from 50 μL to 350 μL depending on pellet size (Figure 3). To maximize EV recovery, rinse the ultracentrifuge tube once with additional 1x PBS and combine with the initial suspension.

### Concentration measurement of EVs by ImageStream

**Timing: 1 h 30 min**

Total and AIT-EV numbers referenced in the protocol are obtained via measurement with the ImageStream system.

The following steps show simple MemBrite staining for overall EV counts.

4. Sample preparation and staining.
  a. For staining, use the 5 μL EVs from Step 3j**.**
  b. Add 5 μL 2× MemBrite 594/615 to achieve a 1x final concentration, vortex.
  c. Incubate for 45 min at 22–25°C in the dark.
  d. During incubation time, filter 1 mL of 1x PBS through a 0.2 μm filter that you will use for the next steps.
5. Purification of the staining reaction.
  a. Fill 450 μL of filtered PBS into a 300 kDa Nanosep filter unit.
  b. Add the solution of stained EVs.
  c. Centrifuge at 5,200 × *g* for 10 min, discard flowthrough.
  d. Recover EVs by pipetting 50 μL of filtered 1x PBS directly onto the Nanosep membrane.
  e. Pipette slowly five times up and down while gently rotating the filter unit.
  f. Transfer resuspended EVs into a 1.5 mL tube.
6. Measure by ImageStream.
  a. Only MemBrite-positive particles are included for analysis and used for concentration calculations. Initial gating during acquisition is performed using side scatter versus the MemBrite fluorescence channel to identify EVs.

***Note:*** For AIT-EV quantification by ImageStream 1 x 10^7^ total EVs are stained with MemBrite 594/615 and fluorochrome-coupled antibodies against human IgG4 and 14-3-3, the previously defined markers for MN-associated AIT-EVs (see troubleshooting problem 3). For AIT-EV quantification by flow cytometry, 1 × 10^6^ total EVs are stained with MemBrite405 and antibodies against human IgG4 and 14-3-3.

For the detailed gating strategy, refer to the Cell publication by Lahme et al. (2026).^5^

**CRITICAL:** MemBrite staining is used as an overall membrane marker for EV detection by ImageStream analysis. However, additional specific antibodies are required to identify distinct EV subpopulations of interest. Since no universally and stably expressed EV protein marker is present at equal levels across all nephrotic urine samples, quantitative analyses should be interpreted comparatively between samples processed in parallel under identical conditions (see troubleshooting: problem 2).

### Enrichment of AIT-EVs from total urinary EVs

**Timing: 16 h**

**Timing: 15 min (step 8)**

**Timing: 15 min (step 9)**

Enrich AutoImmunoglobulin-Triggered EVs (AIT-EVs) from total urinary EV preparations by immunoprecipitation using anti-human IgG4–coupled magnetic DynaBeads. The AIT-EV elution/detachment procedure from the DynaBeads varies depending on further downstream analysis like immunoblot, NTA, immunofluorescence, electron microscopy and mass spectrometry.

***Note:*** The proportion of AIT-EVs within total urinary EVs varies substantially between patients as well as to healthy controls. Absolute AIT-EV numbers cannot be predicted for new samples and should be empirically assessed after the first enrichment round. In our experience, 10^6^ to 10^9^ total EVs can be isolated from 100 mL MN patient urine, yielding approximately 10^4^ to 10^8^ enriched AIT-EVs. For healthy controls the total EV number isolated from 100 ml urine is around 10^4^ to 10^7^, the AIT-EV amount yielding approximately 10^2^ to 10^4^.

**Figure 4:**
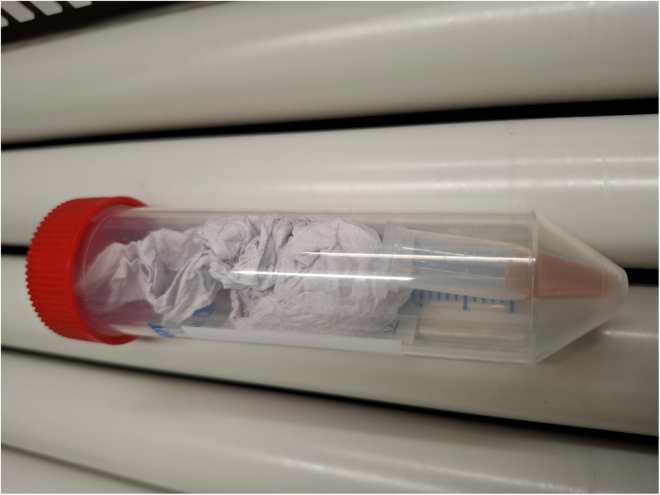
Incubation of DynaBeads (± EVs) on a roller shaker to ensure continuous mixing and to prevent sedimentation
  d. After the overnight incubation, carefully take the 1.5 mL tube out of the 50 mL tube.
  e. Place the 1.5 mL tube on a magnetic rack until you clearly see the DynaBeads gathering on the side of the tube facing the magnet. This usually occurs within seconds (Figure 5).

7. Immunoprecipitation.
  a. Take the remaining EVs from Step 3l.
    **CRITICAL:** If the volume is less than 350 μL, fill up to 350 μL with 1x PBS. For higher volumes, which contain more EVs, you may need to adjust the amount of DynaBeads and coupled antibody.
    ***Note:*** Consider taking an immunoprecipitation „input“ sample of the total urinary EVs that may be retained for comparative analyses. Store at −80°C but avoid long term storage.
  b. Transfer the previously prepared magnetic DynaBeads coupled to the anti-human IgG4 antibody into the EV suspension.
  c. Incubate the EV–DynaBead mixture overnight at 4°C on a roller shaker set to 35 rpm.
    **CRITICAL:** Prevent sedimentation of the DynaBead–EV mixture during incubation. Continuous gentle rotation is essential for efficient binding.
    ***Note:*** To achieve continuous rotation, place the 1.5 mL tube inside a 50 mL tube and stabilize it with a folded tissue so that the small tube remains upright, secure, and does not move during rotation on the roller shaker (Figure 4).

**Figure 5:**
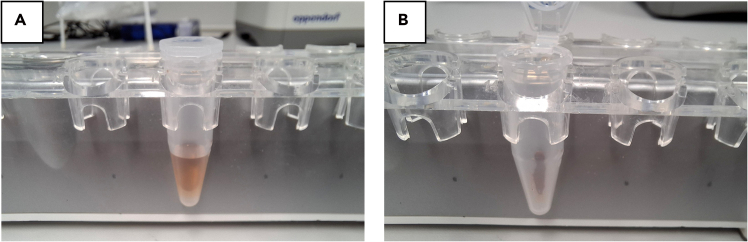
Separation of DynaBeads on a magnetic rack (A) Place DynaBead-EV mixture on the magnet and allow it to stand until the DynaBeads are fully collected at the magnet. Remove the supernatant containing unbound (“rest”) EVs and (B) DynaBeads with bound AIT-EVs remain on the magnet ready for washing.
  f. Remove the supernatant as long as the tube is still placed in the rack, don’t disturb the DynaBeads (Figure 5).
    ***Note:*** The supernatant at this step represents the AIT-EV depleted ‘Rest-EV’ fraction and may be retained for comparative analyses. Store at −80°C but avoid long term storage.
  g. Remove the tube from the magnetic rack.
  h. Add 500 μL of 1x PBS, gently resuspend DynaBeads by slowly turning the tube upside down.
  i. Repeat steps e to g as additional washing steps for the DynaBbeads and the DynaBead-bound AIT-EVs.
  j. Immediately continue with the AIT-EV elution/detachment method of your choice depending on your downstream application, either Step 8 or Step 9.
8. Detachment of AIT-EVs for imaging and particle-based analyses.
  Detach bead-bound AIT-EVs by vortexing to free the AIT-EVs from the beads and resuspend in 1x PBS to measure the concentration by ImageStream and proceed with further analyses.
  ***Note:*** Use this detachment method for immunoblotting, immunofluorescence, electron microscopy, and NTA.
  a. Add 50 μL of PBS to the DynaBeads from Step 7j and vortex thoroughly for 1 min.
  b. Incubate at 37°C for 1 min in a thermoblock, then vortex for another minute.
  c. Place the tube on a magnetic rack until you clearly see the beads gathering on the side of the tube facing the magnet. This usually occurs within seconds.
  d. Transfer the supernatant containing detached AIT-EVs into a new tube.
  e. Repeat Steps a to c**.**
  f. Transfer the supernatant into the same tube used for the first detachment round to obtain a pooled AIT-EV eluate.
  g. Transfer 5 μL detached AIT-EVs into a new tube and continue with Step 4b for concentration measurement by ImageStream.
  h. Use the remaining detached AIT-EVs for downstream applications as soon as possible.
    ***Note:*** Use the following AIT-EV input numbers for downstream analyses:
    Immunoblotting: 1 × 10^4^ AIT-EVs per lane.
    Immunofluorescence: 1 × 10^6^ AIT-EVs per staining panel.
    Electron microscopy:  1 × 10^8^ AIT-EVs per grid.
    NTA:      1 × 10^6^ AIT-EVs.
    Long-term storage of AIT-EVs is not recommended; samples should be used as freshly as possible. In our experience, reliable results can still be obtained when AIT-EVs are stored as a pellet at −80°C for up to 4 weeks.
9. Elution of AIT-EVs for mass spectrometry and elution of AIT-EV bound autoantibodies for immunoblot detection.
  a. Elute the DynaBead bound AIT-EVs with an acidic Elution Buffer to separate the AIT-EVs from the DynaBeads.
    ***Note:*** Use the acidic elution method for proteomic analyses and for eluting AIT-EV-bound antibodies which can subsequently be used as primary antibodies in immunoblotting for autoantibody detection.
  b. Add 50 μL Elution Buffer to the DynaBeads AIT-EV suspension from Step 7j and vortex thoroughly for 1 min.
  c. Incubate at 37°C for 1 min in a thermoblock, then vortex for another minute.
  d. Place the tube on a magnetic rack until you clearly see the DynaBeads gathering on the side of the tube facing the magnet. This usually occurs within seconds.
  e. Transfer the supernatant containing DynaBead-eluted AIT-EVs into a new tube.
  f. Repeat Steps b to d.
  g. Transfer the supernatant into the same tube used for the first elution to obtain a pooled AIT-EV eluate.
  h. Rapidly neutralize samples by adding 100 μL Neutralization Buffer.
  i. Transfer 5 μL eluted AIT-EVs into a new tube and continue with Step 4b for concentration measurement by ImageStream.
  j. Use the remaining eluted AIT-EVs for downstream applications as soon as possible.
    ***Note:*** Use 3 × 10^5^ AIT-EVs per sample for performing proteomic analyses and 10^7^ AIT-EVs for detection of AIT-EV-bound autoantibodies. To separate autoantibodies from AIT-EVs, ultracentrifuge the AIT-EVs from Step 9j at 100,000 x g, 4°C for 70 min. Collect the supernatant which includes the autoantibodies and use as primary antibodies on the immunoblot. Long-term storage of AIT-EVs is not recommended; samples should be used as freshly as possible. In our experience, reliable results can still be obtained when AIT-EVs are stored as a pellet at −80°C for up to 4 weeks.

## Expected outcomes

Using this protocol, AutoImmunoglobulin-Triggered Extracellular Vesicles (AIT-EVs) can be reproducibly enriched from both nephrotic patient urine and healthy control urine. The inclusion of an ultrafiltration step prior to EV isolation is critical for nephrotic samples, as it substantially reduces excess soluble proteins that otherwise interfere with EV recovery, purity, and downstream analyses. Without this step, nephrotic urine yields poorly resolved EV preparations that are unsuitable for reliable molecular or morphological characterization.

Subsequent differential ultracentrifugation enables the isolation of total urinary EVs, while the immunoprecipitation step specifically enriches the AIT-EV subpopulation, resulting in a more purified and biologically defined EV fraction. Application of this workflow typically yields sufficient material for multiple downstream assays, including imaging-based, biochemical, and proteomic analyses.

**Figure 6:**
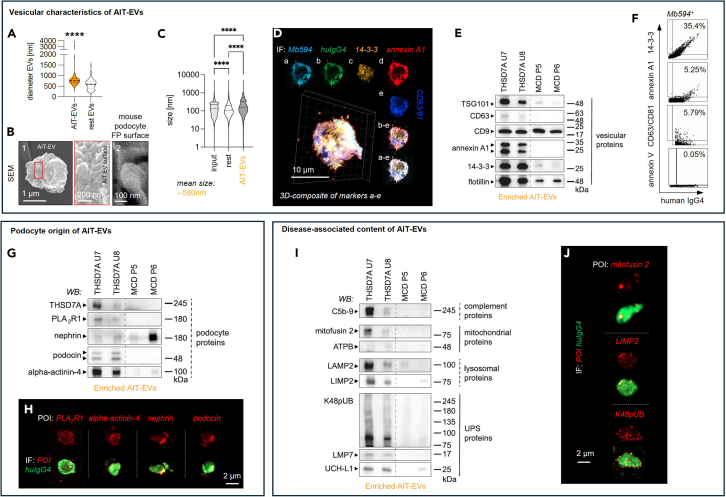
Characterization of AIT-EVs isolated from a THSD7A^+^-MN patient by protein- and particle-based analysis methods (A–F) Vesicular features of AIT-EVs: A) Scanning electron microscopy (SEM) measurement of size distribution demonstrates larger mean AIT-EV size compared to rest EVs (∼858 nm versus 581 nm; ∗∗∗∗*p* < 0.0001, unpaired *t* test). (B) SEM of podocyte-like AIT-EV surface; 50.000×. SEM of mouse podocyte FP for comparison; 100.000×. (C–F) (C) NTA of urinary EV fractions demonstrate increased size of huIgG4-enriched AIT-EVs (∼580 nm; ∗∗∗∗*p* ≤ 0.0001, one-way ANOVA, Kruskal–Wallis). Vesicle marker (14-3-3, annexin A1, CD63/CD81) expression by (D) 3D confocal imaging and (E) immunoblot validation in THSD7A^+^-MN versus minimal change disease [MCD] patients P5/P6. (F) ImageStream confirming abundant 14-3-3 and absence of annexin V (marker of apoptotic bodies) in AIT-EVs. Membrite (Mb)594 stains plasma membrane. (G and H) Podocyte proteins detected in enriched AIT-EVs by G) immunoblot in U7/U8 versus MCD P5/P6 and H) by confocal colocalization to huIgG4. (I and J) Disease-associated proteins (complement: C5b-9; mitochondrial: mitofusin 2, ATPB; lysosomal: LAMP2, LIMP2; ubiquitin proteasome system (UPS): K48pUB, LMP7, UCH-L1) identified in enriched AIT-EVs by I) immunoblot in U7/U8 versus MCD P5/P6 and J) confocal colocalization to huIgG4. Confocal, 630×. Dashed blot lines, non-adjacent bands. POI, protein of interest.

Enriched AIT-EVs obtained using this protocol are expected to display characteristic EV-associated proteins, as confirmed by immunoblotting, ImageStream analysis, and electron microscopy (Figure 6). These analyses support the presence of a distinct EV subpopulation rather than nonspecific protein aggregates. In addition, immunoblotting and mass spectrometry–based proteomics of the enriched AIT-EV fraction consistently detect podocyte-associated proteins and markers known to be upregulated in MN, indicating a podocyte-derived origin of AIT-EVs in this disease context (Figure 6; proteomic data are deposited at the PRIDE repository PXD049008. Accession numbers are listed in the key resources table).

Overall, this protocol enables comparative analyses of AIT-EVs across patient samples and controls and provides a robust foundation for qualitative and semi-quantitative assessment of disease-associated urinary EV subtypes.

In addition, this workflow can be adapted for the enrichment of other EV subpopulations by coupling alternative capture antibodies to the DynaBeads, provided antibody specificity and compatibility with urinary EVs are validated.

## Limitations

Due to the analysis of nephrotic urine samples, complete removal of contaminating soluble proteins is not achievable. Consequently, downstream analyses, particularly highly sensitive techniques, must be interpreted with caution. To increase robustness, findings should ideally be supported by multiple complementary analytical approaches.

For imaging- and antibody-based techniques such as ImageStream analysis, flow cytometry, and electron microscopy, autoantibodies bound to EV-associated antigens may partially mask epitopes. As a result, certain target proteins may not be fully accessible or detectable, depending on the analytical method and antibodies used.

This protocol does not rely on an absolute reference standard sample. Enrichment and analysis of AIT-EVs are therefore inherently relative and primarily qualitative. Comparisons should be performed only between samples processed using identical workflows and conditions. Future adaptations of the protocol may be required as new analytical methods for AIT-EV characterization become available, balancing further reduction of contamination with preservation of EV integrity and surface-associated proteins.

The required starting urine volume for AIT-EV enrichment depends on the urine concentration, the total EV abundance, and the intended downstream analyses. More diluted urine samples generally contain fewer EVs, whereas first-morning urine is typically more concentrated and therefore expected to yield higher EV numbers. Most analyses described here were performed using 24-hour urine collections, and hands-on experience with spot urine samples from patients is currently limited. Based on published data and comparative analyses in healthy controls, major qualitative differences in EV or AIT-EV profiles between spot urine and 24-hour urine samples are not expected; however, a direct systematic comparison in patient cohorts has not yet been performed.

Based on experience with MN patients, a minimum volume of approximately 30 mL of 24-hour urine is typically sufficient for AIT-EV enrichment followed by ImageStream or flow cytometry analyses, and usually also provides sufficient material for immunoblotting. For immunoblot analyses, loading approximately 10^4^ to 1 x 10^5^ AIT-EVs per lane consistently yields reliable signals. Inter-individual variability is substantial, however, and EV yield from the same urine volume may differ markedly between patients. Assessment of urinary creatinine levels may help estimate urine concentration and support interpretation of EV yield.

To minimize variability, the entire 24-hour urine volume is thoroughly mixed upon receipt, aliquoted (typically into 50 mL fractions), supplemented with Preservation Solutions, processed according to the protocol, and stored at −80°C.

Storage in aliquots minimizes repeated freeze-thaw cycles, which negatively affect EV integrity, membrane stability, and overall yield. From a single 50 mL aliquot, two to three downstream analyses can typically be performed, depending on EV abundance and storage duration EV yield and usability strongly depend on storage duration.

For EV demanding applications such as mass spectrometry-based proteomics or electron microscopy, larger starting volumes are generally required (approximately 150 mL). Empirical experience suggests that urine samples stored for extended periods (e.g., 3 years) may require approximately twice the starting volume to achieve yields comparable to relatively fresh samples. As some downstream analyses are performed in batches, urine samples may be stored for different durations prior to analysis, which should be considered during data interpretation.

While modest diurnal variation in urinary EV numbers has been reported, urine concentration, sample handling, storage duration, and freeze-thaw history appear to have a stronger impact on EV yield and quality than the time of day of urine collection. From a practical and translational perspective, spot urine samples are therefore attractive for routine clinical applications; however, further systematic comparisons between spot urine and 24-hour urine samples in patient cohorts are required.

## Troubleshooting

### Problem 1: See ultrafiltration of urine samples

High protein contamination in nephrotic urine interferes with EV purity and downstream analyses.

### Potential solution

Nephrotic urine contains high levels of soluble proteins that can obscure EV-associated signals, particularly in sensitive applications such as mass spectrometry-based proteomics. Multiple strategies were evaluated to reduce protein contamination, including size exclusion chromatography (SEC), dialysis, ultracentrifugation with DTT-containing buffers, iodixanol density gradients, and ultrafiltration.

Among these approaches, ultrafiltration proved most suitable for this protocol, as it effectively reduces soluble proteins while preserving EV surface-associated proteins required for AIT-EV immunoprecipitation and downstream flow cytometric analyses. In addition, ultrafiltration avoids the loss of larger EVs, which was observed with SEC. Based on these observations, ultrafiltration (or, alternatively, dialysis) is currently the preferred method for protein reduction in nephrotic urine samples.

### Problem 2: See 6.a “critical”

Limited availability of stable normalization markers for quantitative AIT-EV analyses.

### Potential solution

Due to high inter-patient variability in nephrotic urine samples, no single EV-associated marker is consistently stable across all samples and conditions. As a result, normalization within individual experiments is challenging, particularly for quantitative analyses.

Rather than relying on a single universal normalization marker, results should be interpreted in a comparative manner by analyzing membranous nephropathy (MN) samples relative to other nephrotic disease groups and/or healthy control samples processed in parallel using identical workflows. This comparative approach reduces the impact of biological variability and enables more robust interpretation of relative changes in AIT-EV abundance or composition.

### Problem 3: See 6.a “note”

High inter-patient variability limits direct comparability of AIT-EV analyses.

### Potential solution

Nephrotic patients exhibit substantial biological variability due to differences in disease etiology, stage, comorbidities, and treatment, which limits direct comparability of isolated and analyzed AIT-EVs. The current marker set (e.g., human IgG4 and 14-3-3) is likely insufficient to fully distinguish AIT-EVs in MN from EV populations present in other nephrotic diseases or in healthy controls, as human IgG4-positive EVs can also be detected in non-MN samples. Future studies should expand flow cytometry or ImageStream staining panels to include additional EV- and podocyte-associated markers. Increasing marker coverage, combined with larger patient cohorts, will facilitate identification of disease-specific marker combinations, improve group stratification, and support the definition of more robust baseline profiles across nephrotic disease entities and controls.

## Resource availability

### Lead contact

Further information and requests for resources and reagents should be directed to and will be fulfilled by the lead contact, Catherine Meyer-Schwesinger (c.meyer-schwesinger@uke.de).

### Technical contact

Technical questions on executing this protocol should be directed to and will be answered by the technical contact, Karen Lahme (ka.lahme@uke.de).

### Materials availability

This study did not generate new unique reagents.

### Data and code availability

The article by Lahme et al. “Autoantibody-triggered podocyte membrane budding drives autoimmune kidney disease,” published in Cell^5^ includes all links to proteomic datasets that were generated and analyzed during this study.

## Acknowledgments

We thank the FACS Sorting Core Unit, the UKE Imaging Facility (DFG RI_00489), and the Nikon Center of Excellence at LIV for excellent technical assistance. All schemes were generated using BioRender. This work was funded by the 10.13039/501100001659Deutsche Forschungsgemeinschaft (DFG), including INST 152/696-3, SFB1192 Project B3, INST 216/1382-1, TRR422 Project B2, ME 2108/10-1, and ME 2108/9-4 (to C.M.-S.). The LSM980 Airyscan 2 was funded by the DFG (INST 152/952-1 FUGG and INST 152/876-1 FUGG).

We thank the patients, Prof. Dr. med Tobias N. Meyer, Dr. med Stephan Segerer, and the team at Barmbek for conscientious sample collection. We are grateful to Prof. Dr. rer. nat. Lars Fester (Nephropathology Bonn) and Michaela Schweizer (UKE) for assistance with AIT-EV electron microscopy, and to the group of Prof. Dr. med Uwe Völker (Genomics Greifswald) for proteomic analyses. We thank Johannes Brand, Lena-Marie Reimers, and Dr. rer. nat. Katrin Neumann for critical reading of the protocol.

## Author contributions

K.L. established and performed all urinary vesicle experiments, including EV isolation, enrichment, immunoblot, ImageStream, NTA, and sample preparation for proteomic analyses. K.L. also performed histological staining procedures, generated micrographs for morphometric analyses, and helped with SEM-based AIT-EV sizing. C.M.-S. conceived the project, designed experiments, and confocal microscopy, analyzed and visualized data, supervised the study. K.L. wrote the manuscript and C.M.-S. jointly streamlined the manuscript.

## Declaration of interests

K.L. and C.M.-S. have an ongoing patent application, PCT/EP2025/056538.
